# Supplementary figures and images for: Temperature and photoperiod differentially impact maternal phenotypes in diapause egg-laying Aedes albopictus mosquitoes
Source: PLoS Negl Trop Dis. 2024 Oct 31;18(10):e0012626. doi: 10.1371/journal.pntd.0012626 (PMC11556710; doi:10.1371/journal.pntd.0012626)

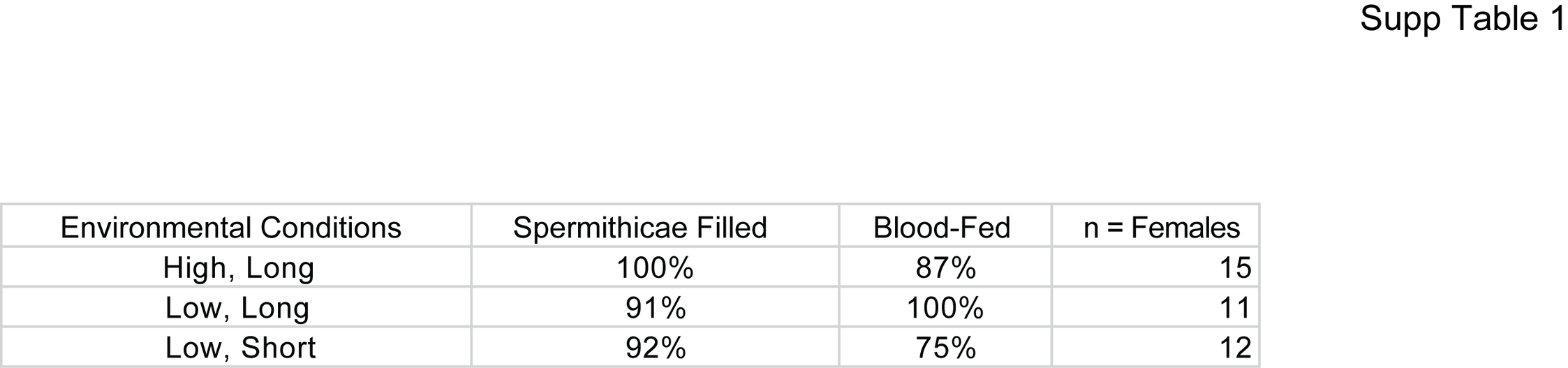

Supplement: S1 Table — Adult females were allowed to mate freely with under three rearing conditions. Female sexual maturation was measured by both the filled spermathecae and ability to blood feed (n = 11–15 females). (TIF) [file pntd.0012626.s001.tif]

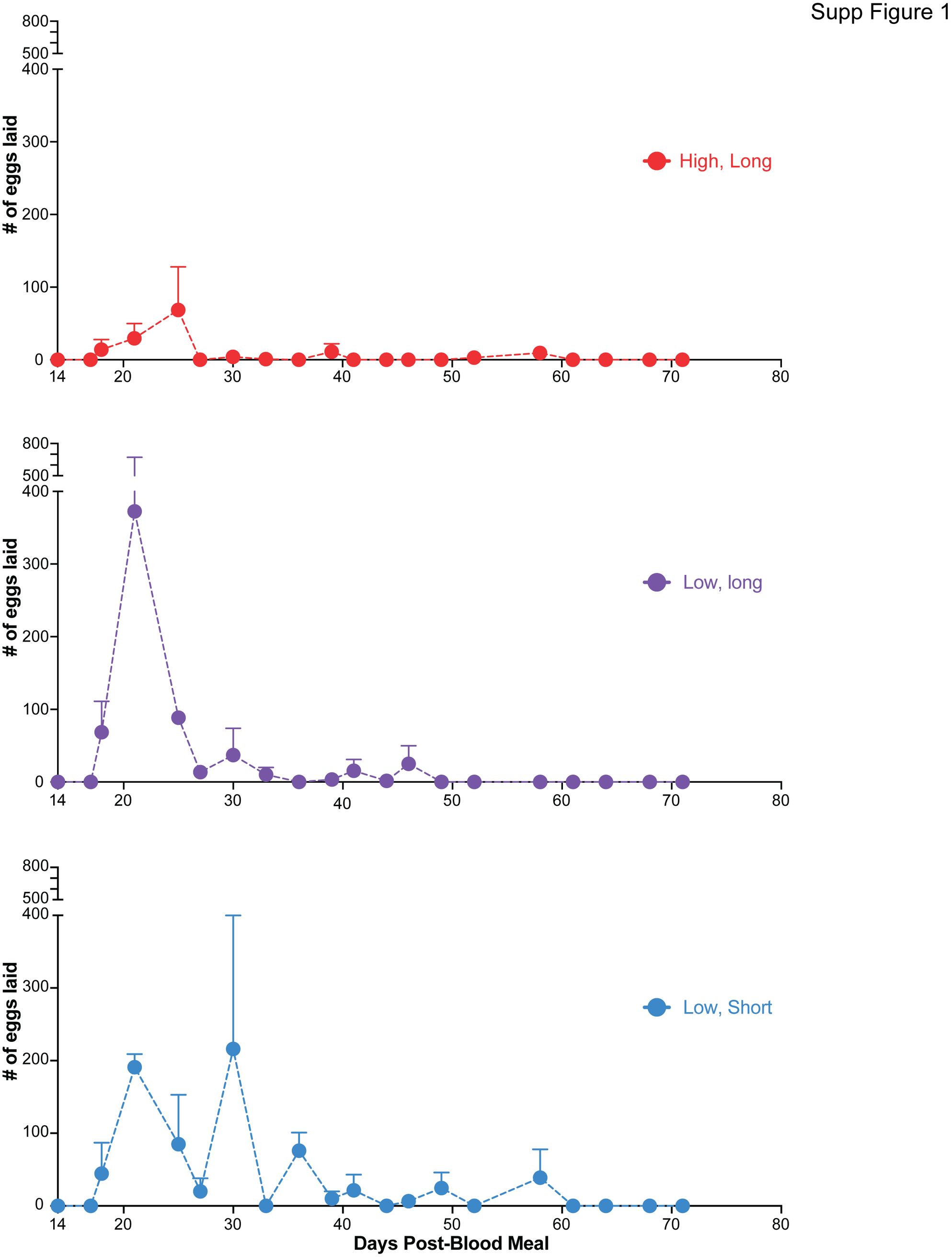

Supplement: S1 Fig — After a single blood meal, oviposition was allowed until death (data is shown as mean with SEM; n = 2 experimental replicates; >23 females per replicate). (TIF) [file pntd.0012626.s002.tif]
